# New insight into the protective effect of *Citrullus colocynthis* loaded with *ZnONP* cream on *glutaraldehyde*-induced dermatitis in health care workers

**DOI:** 10.1038/s41598-024-82066-7

**Published:** 2025-01-09

**Authors:** Walaa A. Ali, Walaa A. Moselhy, Marwa A. Ibrahim, Ahlam G. Khalifa, Gadallah Mohamed

**Affiliations:** 1 Department of Biotechnology and Life Sciences, Faculty of Postgraduate Studies for Advanced Sciences, Beni-Suef University, Beni-Suef, 62511 Egypt; 2 Toxicology and Forensic Medicine Department, Faculty of Veterinary Medicine, Beni-Suef University, Beni-Suef, 62511 Egypt; 3 Department of Biochemistry and Molecular Biology, Faculty of Veterinary Medicine, Cairo University, Cairo, Egypt; 4 Forensic Medicine and Toxicology Department, Faculty of Veterinary Medicine, Beni-Suef University, Beni-Suef, 62514 Egypt; 5 Biochemistry Department, Faculty of Agriculture, Al Azhar University, Cairo, Egypt

**Keywords:** Oxidative stress, Dermatitis, IL-6, PTGFB1, Ptgs2, Nfkb 1, *Citrullus colocynthis*, *ZnONPs*, Anti-inflammatory effects, Biochemistry, Molecular biology, Health care, Health occupations, Medical research

## Abstract

*Glutaraldehyde* (GLU) is mainly used in medicine by healthcare workers during infection control as a chemical disinfectant. It has been linked to numerous health hazards that range from asthma to irritation of the eye to contact dermatitis. *Citrullus colocynthis* (C.C.) is utilized as a supplement to combat a range of health-related problems. This study aimed to assess the effectiveness of locally applied *Citrullus colocynthis* extract and *Citrullus colocynthis* loaded with *ZnONPs* against dermatitis caused by the disinfectant *glutaraldehyde* (2%).The female mice were divided into five groups (G1, G2, G3, G4, and G5). Group 1 was used as a control. The other 4 groups (2,3,4,and 5) were sprayed with 2% *GLU* (2 mg/kg body weight), and the other groups (3,4,and 5) were subjected to local application of natural products (*Citrullus colocynthis* extract cream, *ZnONP* cream, and *Citrullus colocynthis* loaded on *ZnONP* cream), respectively. Each experimental animal was followed for 5 days per week for 30 days.Our findings revealed that GLU-induced dermatitis via the upregulation of TNF-α, IL-1b, NFkb 1, and ptgs2 mRNA expression and the downregulation of TGFB1 mRNA expression caused oxidative stress and altered the biochemical markers and histological appearance. However, these effects were improved by *the ZnONPs*, *C.C.* extract, and *C.C.-ZnONPs*.Local application of *Citrullus colocynthis ZnONPs* and *ZnONPs* had preventive effects against *GLU*-induced dermatitis through the suppression of oxidative stress and inflammatory markers and the enhancement of antioxidants.

## Introduction

Nosocomial infections, or hospital-acquired infections, affect between 5 and 20% of hospitalized patients. It poses a persistent risk to patient safety. Multi-drug-resistant organisms (MDROs) rely on workers to clean and disinfect areas effectively^1^. In addition to social and economic hazards MDRO are key variables that can lead to nosocomial infections and increase the cost of medical care^2^. Outbreaks of nosocomial infections are usually caused by contaminated tools and other medical supplies^3^. Disinfection is considered one of the best tactics for sterilizing reusable instruments^4^. Disinfectants can lower the number of microorganisms in the environment and obstruct their path of transmission^5^. Chemical disinfectants are the most popular and widely utilized type of disinfectant^6^. One of the most commonly used groups is the aldehyde group. *Glutaraldehyde*is now widely used as a chemical sterilant and high-level disinfectant. Agent^7^. *Glutaraldehyde*is employed in the dental and medical fields^8^. It has been linked to a number of dangerous health outcomes, including asthma, breathing difficulties, irritation of the eyes, and contact dermatitis^9^. Contact dermatitis has been observed in X-ray technicians, endoscopy nurses, dental assistants, hospital maintenance and cleaning personnel, and funeral service workers^10^. Synthetic drugs used to treat dermatitis caused by *GLU* have certain side effects, such as hypersensitivity. Natural products are preferred over synthetic products because they have fewer or no negative effects, are more affordable, and are more readily available. One of these natural products is *Citrullus colocynthis*. The nutrients present in *Citrullus colocynthis*may be taken as supplements to help with a variety of health-related problems^11^. It has many antibacterial, anti-inflammatory, and antioxidant properties^12^. *Citrullus colocynthis*has been linked to a protective effect by suppressing proinflammatory release, inhibiting COX-2 expression, and lowering both TNF-Ì` and IL-6 levels. Nanomedicine is the use of nanoparticle technology for the treatment and prevention of illnesses in humans. The second most prevalent element in the human body, controls several forms of metabolism, cell growth and proliferation, and the creation of macromolecules such as DNA, RNA, and proteins^13^. *Zinc* impacts the synthesis of cytokines that reduce inflammation, including interleukins such as IL-1b, IL-6, and TNF-a (tumor necrosis factor alpha). compared to other NPs ,zinc oxide nanoparticles significantly reduce inflammation because of their high photocatalytic efficiency, affordability, and lack of toxicity. Therefore, our experimental research was carried out to assess the effectiveness of locally applied *Citrullus colocynthis* extract and *ZnONPs* loaded with *Citrullus Colocynthis* and *ZnONPs* to prevent dermal inflammation when a chemical disinfectant (*glutaraldehyde* 2%) was used.

## Materials and methods

### Plant materials

Dryied *Citrullus Colocynthis* (fruit) raw samples were purchased from a medicinal herb dealer in Helwan, Cairo, Egypt.

*Citrullus colocynthis* (fruit) identification number 0E49E3V9U6.

### Chemicals

1.*Glutaraldehyde *(2.2% solution) was obtained from Sigma Chemical Industries Company.

2.*Zinc oxide* purity: 99.5% w/w from the Nanotechnology Laboratory, Faculty of Postgraduate Studies for Advanced Sciences (PSAS), Beni-Suef University.

### Preparation of *Citrullus Colocynthis (C. C)* extract

**Fig. 1:**
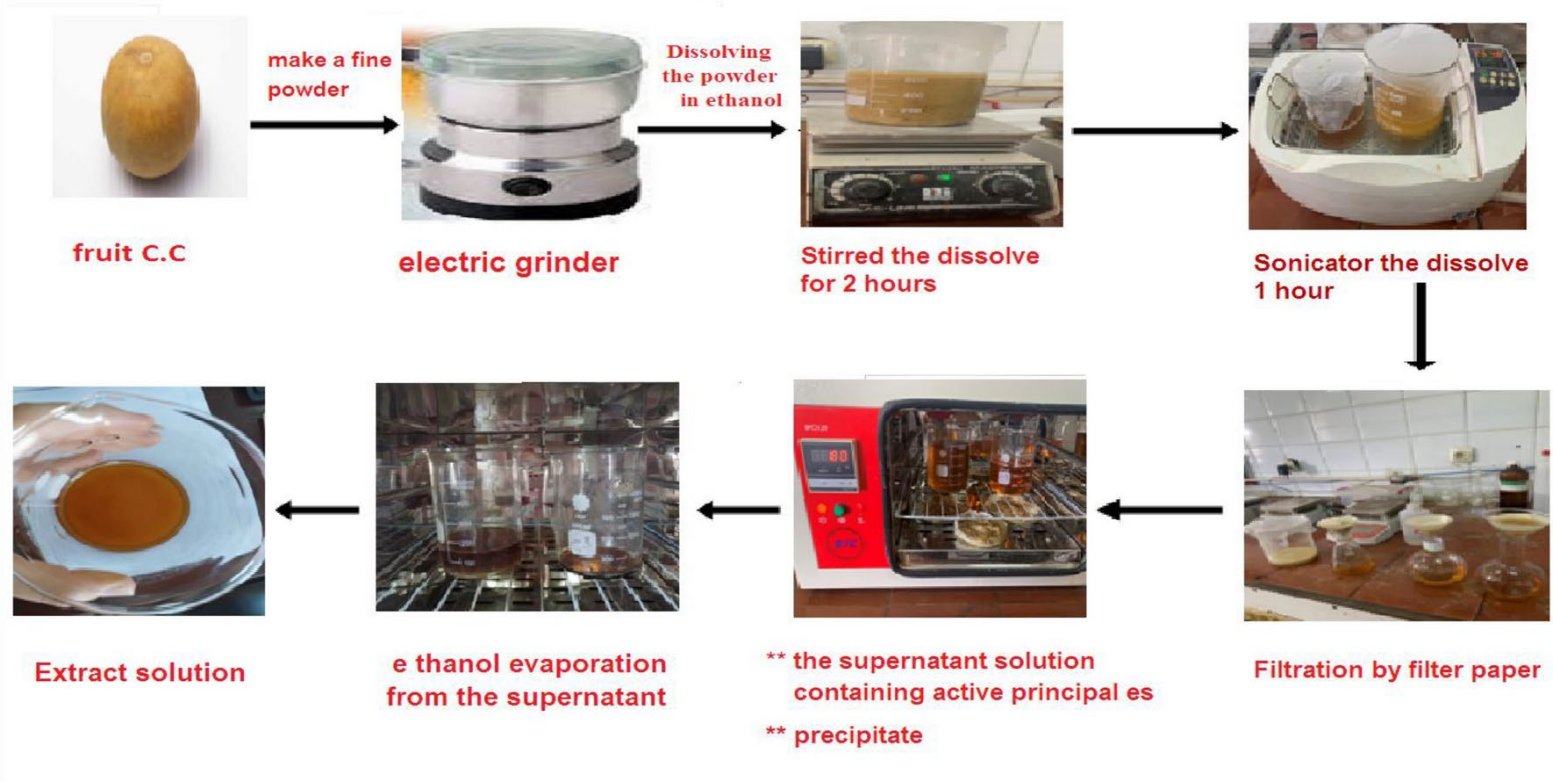
Preparation of Citrullus Colocynthis (C.C) Extract.

Fruits of *C.C.*extract were created, according to^14^ (Fig. 1).

### Synthesis of *Zinc Oxide* nanoparticles (ZnONPs)

*ZnONPs*were created according to the protocol of^15^. Zinc oxide (ZnO) was dissolved in 100 ml of deionized water for 40 min and agitated in the solution until it was entirely dissolved. Sodium hydroxide solution was added progressively over 3 h with vigorous stirring until a white precipitate formed. The solution was recovered by centrifugation at 15,000 rpm for 10 min. The precipitate was then filtered and thoroughly washed with deionized water before being dried in an oven at 70°C for 48 h until completely dry.

### Loading of *Citrullus Colocynthis* on ZnONPs (C.C-ZnONPs)

**Fig. 2:**
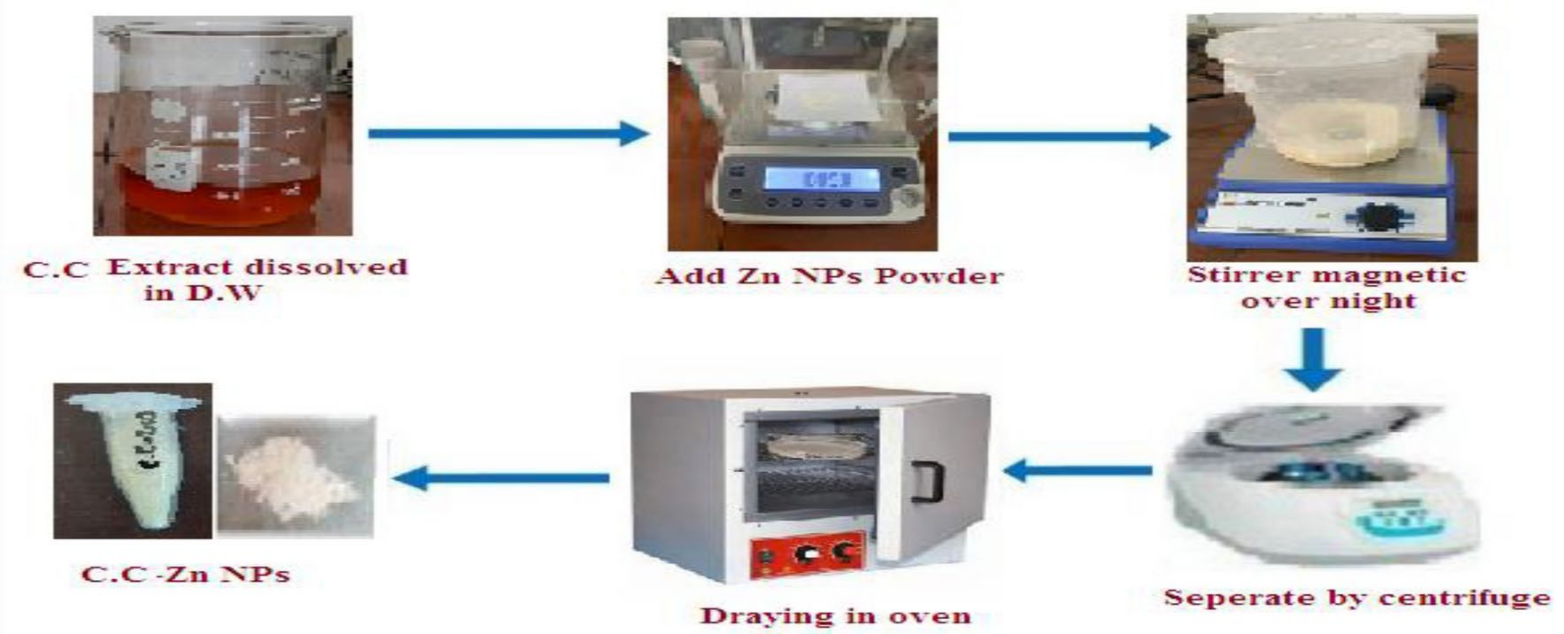
Loading of Citrullus Colocynthis (C.C)on ZnONPs.

*Citrullus colocynthis*extract was loaded onto ZnONPs via the coprecipitation method of^16^. The supernatant was removed. The formed *C.C-ZnONPs*were dried at 60 °C for 4 h in an oven^17^. During the drying period, the Zn (OH)2 in the NPs fully converted into white *ZnONP* powder. *ZnONPs* and *ZnONPs/C.C.* will be characterized via ultraviolet visible (UV) spectroscopy, Fourier transform infrared (FTIR) spectroscopy, and transmission electron microscopy (TEM). (Fig. 2).

### Experimental design

Forty healthy adult female mice, seven weeks old, were housed in eight rats per cage. The mice were obtained from the National Research Center in Dokki. All procedures for the maintenance and sacrifice (care and utilization) of the animals were performed. The empiricism processes were carried out in compliance with the ethical procedures and standards that were authorized by Beni-Suef University’s local animal ethics committee (permission from Ethics Committee No.022–383). The mice were divided into five groups, each consisting of eight mice. Group 1 (control), no exposure to *GLU*or creams Group 2 (GLU) exposed GLU spray (an applied volume of 2.0 ml/kg/day) from^18^. Group 3 *(GLU* + *ZnONPs)* was exposed to both *GLU* spray and *ZnONP* cream. Group 4 *(GLU* + *C.C.* extract) was exposed to both *GLU* spray and *C.C.* cream. Group 5 *(GLU* + *CC-ZnONPs)* was exposed to both GLU spray and *C.C-ZnONP* cream. Throughout the trial, the animals in each group underwent daily examinations, and clinical indicators were monitored. At the beginning of the study, the hair of the mice was shaved on the dorsal skin surface, and then the mice were exposed to *GLU* spray (four groups) with the exception of the control group. The cream was applied 3 h after *GLU*. All mice were euthanized at the conclusion of the 30-day experiment by isoflurane inhalantion (300 µL on gauze placed in a 500 ml container). The skin tissue was meticulously split into three sections according to the protocol of^19^.

### Biochemical assays

The levels of glutathione (GSH) were estimated via an ELISA kit via sandwich-ELISA as described previously (Catalog No. SL0998Ra, Bioassay Technology Lab) and malondialdehyde (MDA) were measured via a spectrophotometric instrument to ascertain the degree of inflammation and the quantity of MDA chemical generated viathe lipid peroxidation process. A commercial MDA ELISA Kit (MDA Assay Kit competitive ELISA) was used for this investigation (catalog no. ER1878, Bioassay Technology Lab).

### Histopathological investigations of skin tissue

The dorsal skin of animals was freshly isolated, and the samples were fixed and stained with hematoxylin and eosin following the protocol of^20^.

### Relative mRNA expression analysis of TNF-α, IL-1b, nfkb1, ptgs2 and tgf1b via qRT-PCR

A Qiagen Mini-RNAeasy extraction kit was used to extract total RNA in accordance with the manufacturer’s instructions. The RNA sample concentration and purity were assessed via spectrophotometry at wavelengths of 260 and 280 nm^21^. To remove any DNA contamination, DNase I treatment was performed. The Revert Aid First Strand cDNA Synthesis Kit from Thermo Scientific was subsequently used to generate complementary DNA (cDNA) in accordance with the manufacturer’s instructions^22^.

The primer sets used for determining the target genes’ mRNA levels were designed on the basis of *Mus musculus*sequences obtained from GenBank. The primer3 tool was utilized to create these primer sets. SYBR Green PCR Master Mix (catalog number: 4,309,155) and ABI Prism Step One from Thermo Scientific were used for real-time PCR measurement of relative gene expression. An instrument from Applied Biosystems was used, according to the manufacturer’s guidelines. For each sample, two PCRs were run^23^. The expression of the housekeeping gene beta-actin was used to standardize the expression levels of the target genes. The primer sets used for detecting the mRNA levels can be found in Table 1.

**Table 1:** Primer sequences of the target genes.

*Gene symbol*	Gene description	Accession number	Primer Sequence	Amplicon size
*TNF*-α	Tumor necrosis factor	NM_013693.3	F: 5′‐ TGTAGCCCACGTCGTAGCAA ‐3′ R: 5′‐ ATAGCAAATCGGCTGACGGT ‐3′	216
*IL-1β*	Interleukin 1 beta	NM_008361.4	F: 5′‐ TGCCACCTTTTGACAGTGATG −3′ R: 5′‐ AAGGTCCACGGGAAAGACAC −3′	220
*NFkb1*	nuclear factor kappa	AY521463.1	F: 5′‐ CCCTACGGAACTGGGCAAAT −3′ R: 5′‐ TGCAAATTTTGACCTGTGGGT −3′	241
*PTGS2*	prostaglandin-endoperoxide synthase 2	NM_011198.5	F:5’-CATCCCCTTCCTGCGAAGTT-3’ R:5’-CATGGGAGTTGGGCAGTCAT-3’	178
*Tgf1b*	Transforming growth factor 1 beta	NM_011577.2	F: 5’- ACTGGAGTTGTACGGCAGTG-3’ R:5’- GGGGCTGATCCCGTTGATTT-3’	123 111
*ACTB*	Beta actin	NM_007393.5	F:- 5′- CCACCATGTACCCAGGCATT −3′ R:- 5′- AGGGTGTAAAACGCAGCTCA-3′	253

### Statistical techniques

The statistical program at Social Science (IBM Corp., released 2017) was used to examine the data. Armonk, NY: IBM Corp.; IBM SPSS Statistics for Windows, Version 25.0. A P-value of less than 0.05 was considered significant.

## Results

### UV–VIS spectrophotometer

**Fig. 3:**
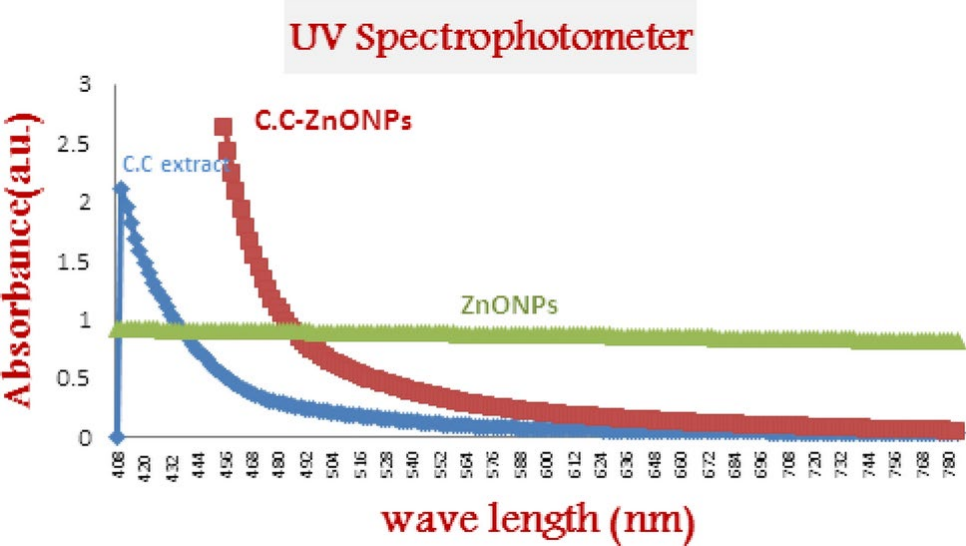
UV–Vis spectra of ZnONPs, C.C extract, and C.C- ZnONPs.

UV–VIS spectroscopy was employed to verify the *C.C.ZnONPs*, as shown in Fig. 3. The UV–VIS spectra showed a narrow peak at approximately 408 nm, which was attributed to adsorption of bimolecules. These absorptions are related to transitions, which correspond to the existence of polyphenolic substances found in the extract. For the *C.C.ZnONP* sample, a narrow peak with a 456 nm absorption band is characteristic of* ZnONPs* (Fig. 3).

### Fourier transform infrared spectroscopy (FT-IR) analysis

**Fig. 4:**
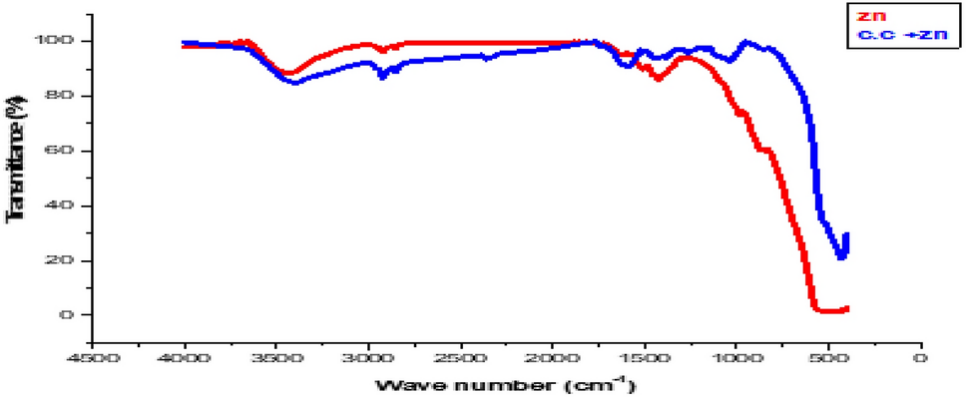
FTIR spectra of zinc oxide nanoparticles and Cc − ZnONPs.

The FTIR analysis of synthesized zinc oxide nanoparticles (Fig. 4) revealed that the peak, 1421.95 cm-1, corresponded to the O–H groups of alcohol. The transmigration of the halo compounds (C–Cl or C–Br stretching) is indicated by the well-defined signal at 1623.19 cm1. The medium peak (1623.19 cm − 1) reflected the C–C = C symmetric stretching of the conjugated alkene groups (1-propane).

The transformation caused by the N = C = S stretching of the isothiocyanate group is responsible for the peak at 1508.60 cm − 1. Propyne (HC ∕ C-CH3) has an alkyne group present, and carbonic dioxide exhibits O = C = O stretching with a prominent peak at 2962.25 cm − 1. The presence of N–H stretching from primary and secondary amine groups is confirmed by the peak at 3444.08 cm1. The alcohol group is visible at 3444.08 cm1 due to O–H vibration. *Citrullus colocynthis* alkaloids, flavonoids, glycosides, phenols, saponins, tannins, and terpenoids are all present in this fruit extract. These phytochemicals contain compounds that increase the stability of the *C.C.-ZnONP* molecule, which was subjected to FTIR analysis (Fig. 6a), revealing that the *ZnONP* crystal structures produce wave lengths at different values and that some phytochemicals have been adsorbed onto the surfaces *ZnONPs.* At 3405.68 cm1 for -OH, 2922.81 cm1 for -C-H, and 2852.53 cm1 for -OH, the vibrations for -C≡C-, 1584.47 cm1 for -N–H, 1430.02 cm1 for -COOH, 1035.00 cm1 for -C-F, 1264.32 cm1 for C–O–C, 1169.99 cm1 for C-H, 464.79 cm1 for C-S, and 429.74 cm1 for C–Br, respectively. Figure 4.

### Transmission electron microscopy (TEM) observations

**Fig. 5:**
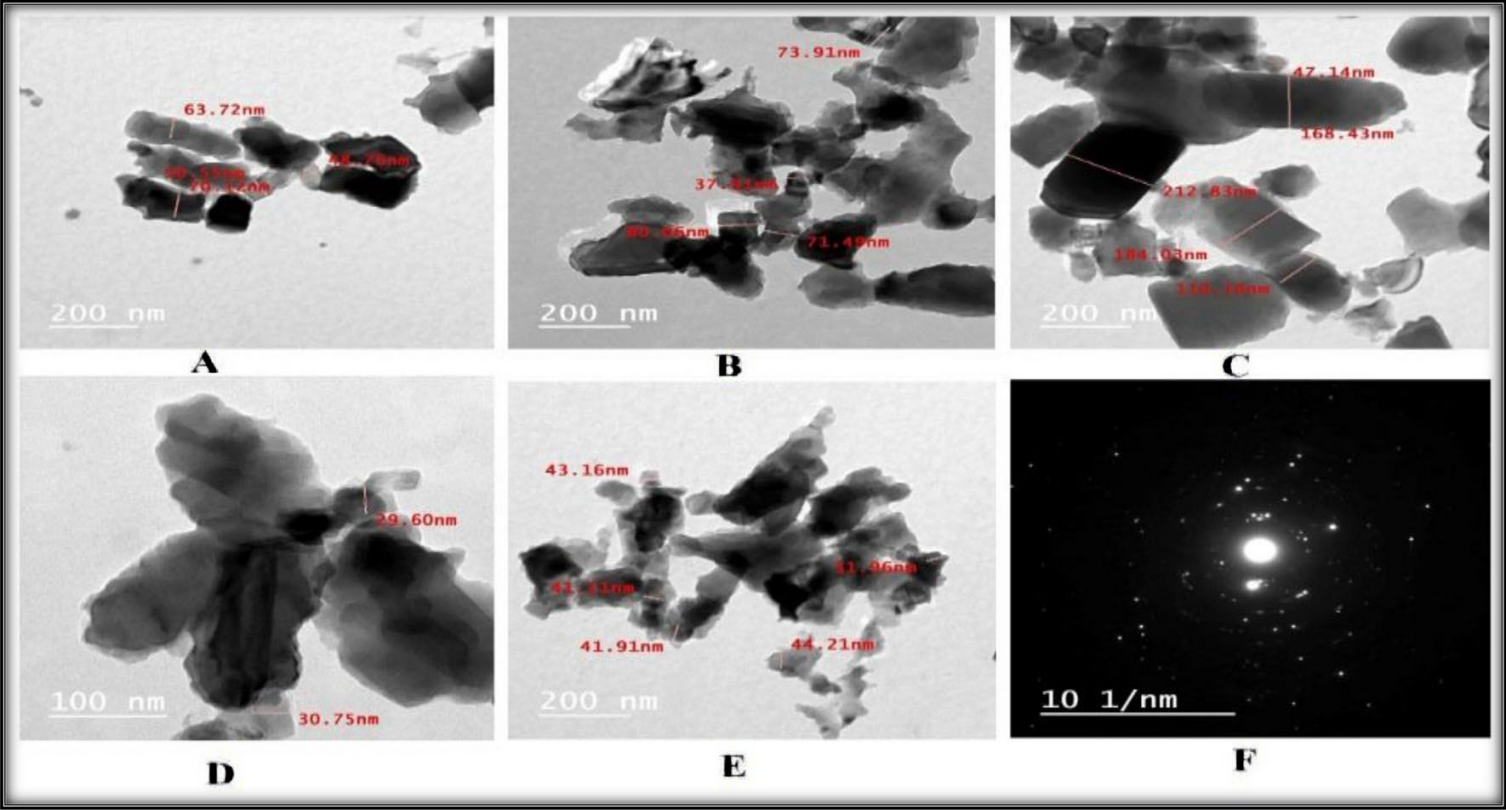
TEM images of C.C-ZnONPs.

Figure 5 shows the *C.C-ZnONP* flower-shaped TEM image. The TEM image displayed here demonstrates that the nanoparticles formed an assembly of asymmetric polygons (Fig. 5).

### Biological assay

The levels of MDA and GSH in skin tissue varied significantly among the groups under study Table 2:

**Table 2:** Biological parameter in skin tissues among studied groups.

		Control	Glu	GLU + ZnONPs	GLU + C.C extract	GLU + CC-ZnONPs
GSH	Mean	167.200	46.1667	125.3000	103.6933	116.4000
	SD	0.10000^e^	0.05774^a^	0.10000^d^	0.09609^b^	0.26458^c^
MDA	Mean	9.1500	41.8067	15.5667	23.5333	21.4667
	SD	0.1000^a^	0.04041^e^	0.15275^b^	0.07234^d^	0.05774^c^

Compared with that in the control group, the level of the antioxidant enzyme (GSH) in the *GLU* treated group significantly decreased (P ≤ 0.001), but this was also linked to a large increase (P < 0.001) in the quantity of MDA.

Compared with those in the *GlU* exposure group, the level of the antioxidant enzyme (GSH) (P < 0.001) significantly increase, and the amount of MDA. (P ≤ 0.001) significantly decrease *ZnONP-*treated group.

The *Citrullus colocynthis*-treated groups were compared with those in the *GLU* and control groups, there was a substantial increase in the GSH level (P < 0.001), which was correlated with a reduction (P ≤ 0.001) in the MDA concentration.

The GSH level was significantly elevated (P < 0.001) in the *C.C.-ZnONP*-treated groups, which was correlated with lower MDA concentrations than those in the *GLU and Citrullus colocynthis* groups.

### Histopathological findings

**Fig. 6:**
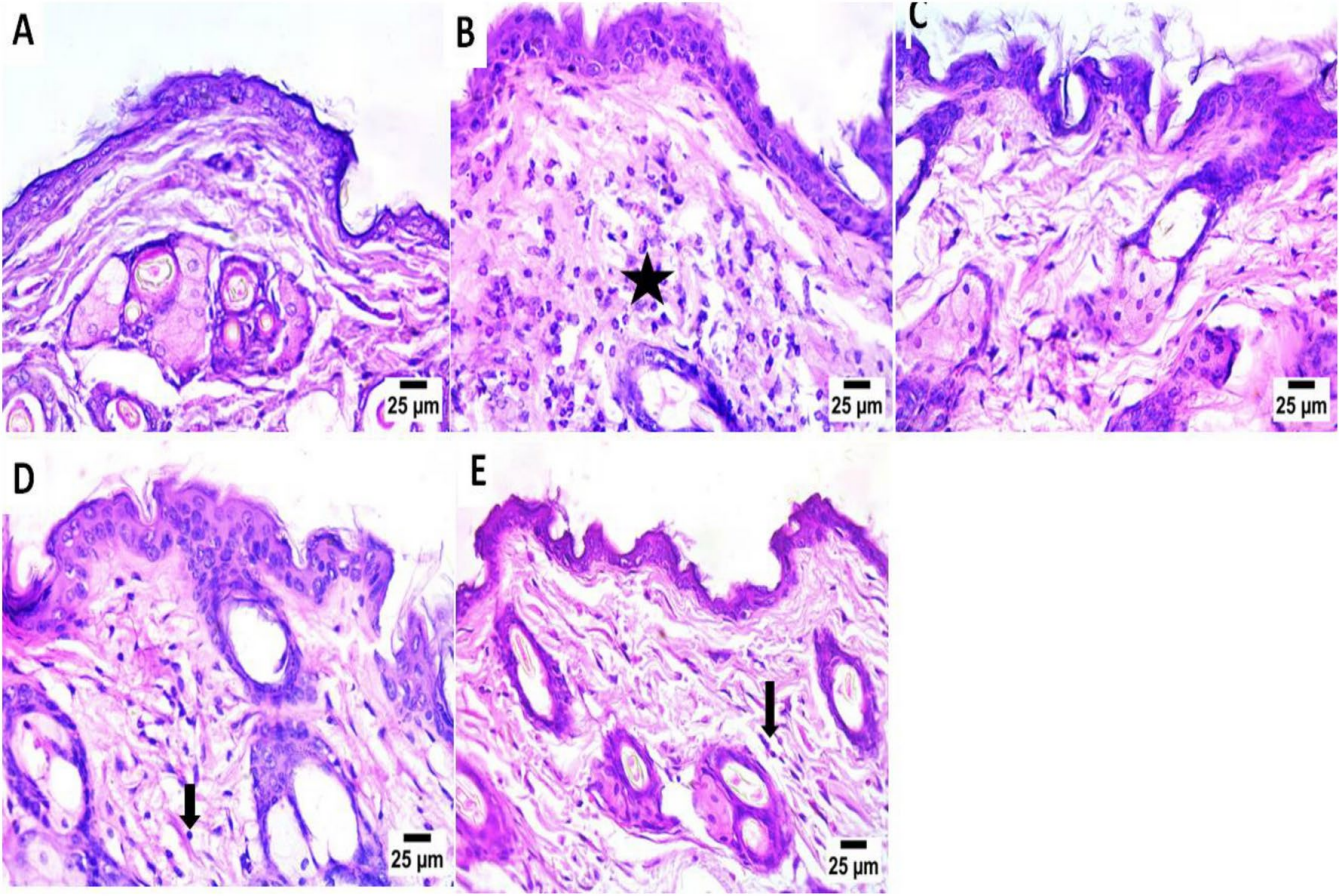
Histological sections of skin from the mice Scale bar = 25 µm (H& E stain). (**A**) control, (**B**) GLU, (**C**) GLU + ZnONPs (**D**) GLU + C.C extract and (**E**) GLU + CC-ZnONPs.

A shows hematoxylin and eosin (H&E) staining of skin tissues from the different groups. At 30 days after treatment (Fig. 6).

Inflammation was still severe in the *glutaraldehyde* groups, with a photomicrograph showing infiltration of the epidermis and dermis by a high number of inflammatory cells, mainly neutrophils and eosinophils (star), with congestion of dermal blood vessels (arrowhead) (Fig. 6B).

In addition, the healing process began in the groups treated with *GLU* + *ZnONPs* and the control groups as shown by photomicrographs of the normal histological structure of the epidermis and dermis Fig. 6(A, C).

Inflammation and allergies were low in areas of the skin in the *GLU* + *C.C* extract photomicrograph shows dermal infiltration by a low number of mononuclear inflammatory cells (arrow), as shown in Fig. 6(D).

The relative reduction in *GLU* + *CC-ZnONP* photomicrograph shows dermal infiltration by a low number of eosinophils (arrow) (Fig. 6E).

### Relative mRNA expression analysis of TNF-α, IL-1b, nfkb1, ptgs2 and tgf1b via qRT-PCR

QRT-PCR was employed. Compared with the control group.

*GLU* exposure resulted in a significant downregulation of TGFB1 mRNA expression and an upregulation of TNF-α, IL-1b, nfkb 1, and ptgs2 mRNA expression.

*Citrullus colocynthis* extract cream alone downregulated TNF-α, IL-1b, NFkb1, and ptgs2 gene expression and upregulated TGFB1 mRNA expression compared with that in the *GLU* group.

Compared with the *GLU* group , *C.C-ZnONP* cream treatment downregulated TNF-α, IL-1b, NFkb1, and ptgs2 gene expression and upregulated TGFB1 mRNA expression levels.

Compared with the *GLU* group, the *ZnONP* cream alone significantly downregulated the TNF-α, IL-1b, NFkb1, and ptgs2 gene expression levels and upregulated in TGFB1 mRNA expression levels.

**Fig. 7:**
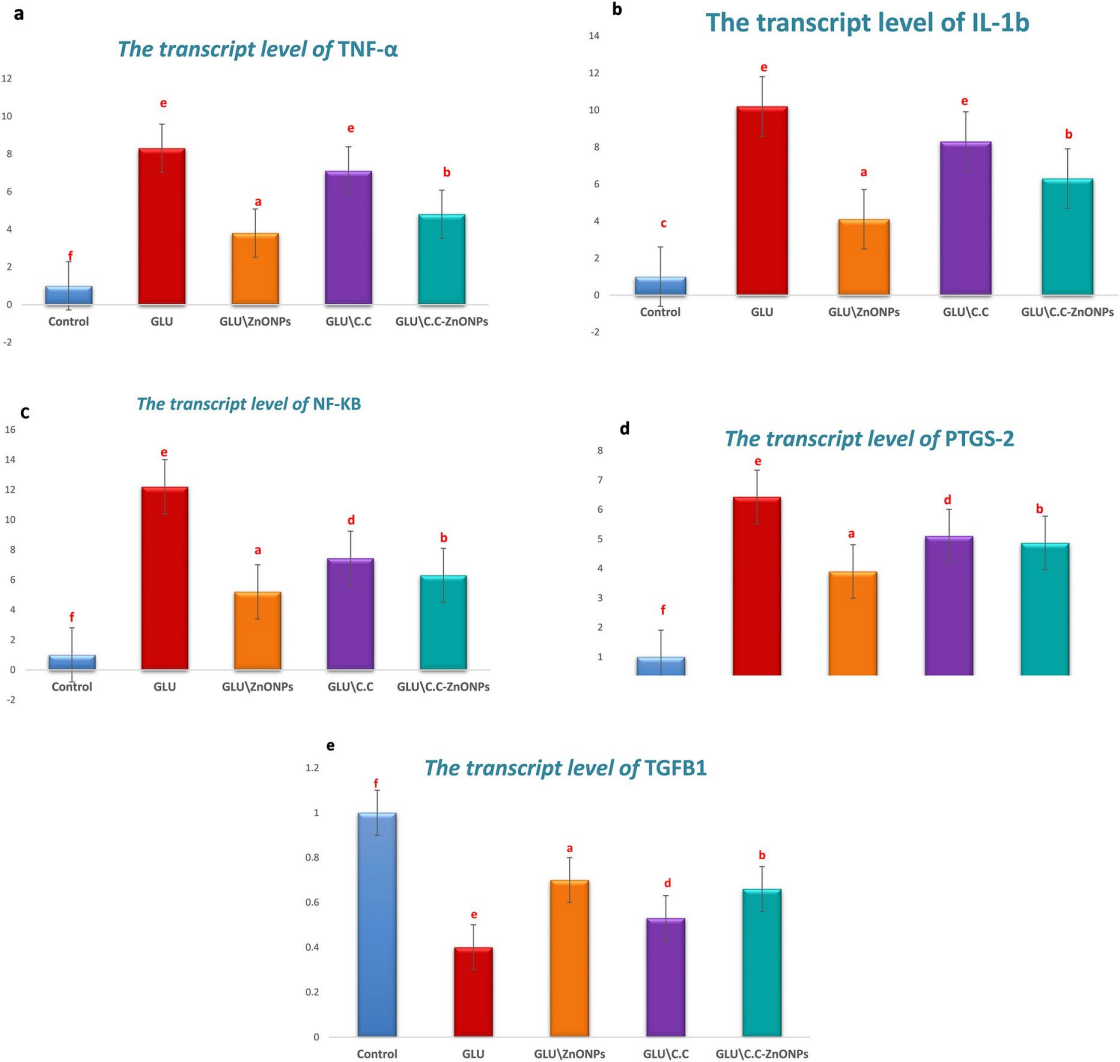
The transcript levels of (**a**) tnf-ɑ; (**b**) il1b; (**c**) nfkb; (**d**) ptgs-2; (**e**) tgf1b genes from different groups. Values expressed as mean ± SE, different superscripts indicate significant distinctions at P < 0.05 (n = 7).

Figure 7 displays the mRNA expression profiles.

## Discussion

Health care workers (HCWs) are mostly exposed to chemical compounds such as disinfectants, sterilizers, and cleaners, leading to a spectrum of healthcare burdens, such as dermatological and respiratory side effects^24^.

*Glutaraldehyde* is considered one of these chemical disinfectants, and can lead to dermatitis. Our study highlights the prevention of dermatitis and any side effects to the skin through the use of using medicinal plants. This material has great potential for therapeutic applications.

*C. colocynthis*is one of the most, common biologically active chemicals, and has different phytoconstituents, including phenols and flavonoids^25^. In the present study, our extract was generally rich in these compounds (alkynes, hydrocarbons, phenols, hydroxyl (OH), aromatics, carboxylic acids, nitro compounds, fatty acids, aldehydes, alkyl alides , cycloalkanes, C–O–C, C-H, C-S, and C–Br), ehich were identified via the FT-IR analysis which determines the wavelength of the compound (Fig. 4). These results align with those of previous studies^14^. These compounds are most likely related to the antioxidant and anti-inflammatory properties of *Citrullus colocynthis*^26^. The Loading of *C.C*. extract on the *ZnONPs* was studied via UV–Vis spectra, TEM,and FTIR spectroscopy to confirm the successful formation and stability of the *CC-ZnONPs*.

The characterization results of the UV–Vis spectra revealed characteristic absorption peaks at 408 nm (Fig. 3)^27^**.** The TEM morphological analysis of *CC-ZnONPs* revealed that they were flower-shaped, and the image displayed here demonstrated that the nanoparticles formed an assembly of asymmetric polygons (Fig. 5)^28^. FTIR spectroscopy confirmed the loading of the extract on the *ZnONPs*^29^.Compared with the conventional methods, nanoparticle administration methods have many advantages (large surface area to volume ratio, greater efficacy, greater absorption)^30^.

The current study shows the efficacy of the local application of *Citrullus colocynthis—ZnONPs* for the prevention and treatment of dermatitis induced by *glutaraldehyde*. Through different methods (biochemical, histological and molecular methods).

Biochemistry studies have revealed the expression of antioxidant (GSH) and oxidative damage related malondialdehyde (MDA), which are highly toxic compounds^31^.

*Glutaraldehyde*exposure results in significantly increased (MDA), and decreased GSH, indicating long-term stress exposure lead to oxidative stress, this result align with those of previous studies^32^.

Our results revealed that *Citrullus colocynthis*extract alone, a minimal decrease (MDA) in these results aligns with those of previous studies^33^and a minimal increase in the GSH content. These results align with those of previous studies^34^. Compared with *Citrullus colocynthis*—*ZnONPs* significantly increase GSH and decrease MDA, indicating that *Citrullus colocynthis*-*ZnONPs* are more effective and more protective, compare with *Citrullus colocynthis* alone.

In the *ZnONP*- treated group, the MDA content decreased^35^, and there was an exponential increase in the GSH level, indicating low stress exposure and increased protection.

Histopathological examination revealed that *glutaraldehyde*resulted in infiltration of the epidermis and dermis by a high number of inflammatory cells, mainly neutrophils and eosinophils, with congestion of dermal blood vessels. These findings indicate severe inflammation, which align with the findings of previous studies^36^.

Photomicrographs of *ZnONPs* revealed a normal histological structure of the epidermis and dermis where *ZnONPs*have the ability to modulate the degranulation process in neutrophils to protect skin tissue^37^^,^^38^.

Photomicrographs of *Citrullus colocynthis* alone revealed that a dermal infiltration by a low number of mononuclear inflammatory cells, compared with that caused by *glutaraldehyde.*

A photomicrograph of *C. colocynthis-ZnONPs* revealed that a dermal infiltration by a low number of eosinophils, there is no mononuclear inflammatory cells, unlike that caused by *Citrullus colocynthis* alone. The anti-inflammatory effect of *C. colocynthis-ZnONPs* was greater than that of *Citrullus colocynthis*alone. These results align with those of previous studies^39^. These NPs improve drug delivery and increase the bioavailability of phytoconstituents adsorbed on them because of their small size, large surface area, and high adsorption of bioactive phytochemicals from *Citrullus colocynthis*^40^.

Molecular examination revealed that *glutaraldehyde*exposure significantly upregulated TNF-α, IL-1b, NFkb 1, and ptgs2 mRNA expression, and downregulated TGFB1, these results are consistent with those of^41^. This leads to the increase in immune cells (serotonin, histamine, and prostaglandin) therefore, the ability of* glutaraldehyde* to induce dermatitis.

*Citrullus colocynthis*extract alone downregulated TNF-α, IL1b, NFkb 1, and ptgs2 gene expression and upregulated TGFB1 mRNA expression. These results align with those of previous studies^42,43^. *Citrullus colocynthis*inhibits the activity of Cox2 and the production of prostaglandins thus reducing inflammation and pain^44^.

*C. colocynthis- ZnONPs* downregulated TNF-α, IL1b, NFkb 1, and ptgs2 gene expression and upregulated TGFB1 (anti-inflammatory mediator) to a greater extent than did *Citrullus colocynthis* only, indicating that *C. colocynthis- ZnONPs* are more effective and more protective.

*ZnONPs*significantly upregulated TGFB1 mRNA expression, and these results align with those of previous studies^45^and downregulated TNF-α, IL1b,NFkb 1, and ptgs2 gene expression through nuclear factor-κB (NF-κβ) pathway inhibition^46^, and the inhibition of the degranulation of mast cells^47^. Which prevents the release of serotonin and histamine. Our results revealed that the anti-inflammatory impact of the *ZnONP* group was greater than that of the *C. C-ZnONPs* group ,which has a much more potent anti-inflammatory effect than the *C. colocynthis -ZnONPs* , which acts through different pathways (prostaglandin and kinin-like pathways), and these findings need further study.

## Conclusions

Local application of natural products loaded with nanoparticles (*Citrullus colocynthis-ZnONPs*) can mitigate *glutaraldhyed*-induced dermatitis by decreasing inflammatory markers and oxidative stress.

## Data Availability

All the research data will be available on request from the corresponding author.

## References

[1] Sottani, C. et al. Effectiveness of a combined UV-C and ozone treatment in reducing healthcare-associated infections in hospital facilities. *J. Hosp. Infect.* **139**, 207–216. 10.1016/j.jhin.2023.06.029 (2023). 37478911 10.1016/j.jhin.2023.06.029

[2] Sahlabadi, F. et al. The effectiveness evaluation of current disinfectants on pathogens isolated from surface of different parts of Shahid Sadughi accidents burns Hospital in City of Yazd. *J. Environ.* **3**(2), 93–101 (2016).

[3] Gill, S.A., Gill, S.H.P. Port site infection due to atypical mycobacteria after laparoscopic surgery in tertiary care hospital of north India*. J. Cardiovasc. Dis. Res.* **14** (05), ISSN:0975-3583,0976-2833 (2023)

[4] Lin, W. et al. Toxicity and metal corrosion of glutaraldehyde-didecyldimethylammonium bromide as a disinfectant agent. *J. Hindawi BioMed Res. Int.* **2018**, 9814209. 10.1155/2018/9814209 (2018). 10.1155/2018/9814209 PMC6069694 30079353

[5] Chen, Z. et al. Effective elimination of bacteria on hard surfaces by the combined use of bacteriophages and chemical disinfectants. *Microbiol. Spectrum* **12**(4), e03797-23 (2024). 10.1128/spectrum.03797-23 PMC10986474 38483478

[6] Aranke, M. et al. Disinfectants in interventional practices. *J. Curr. Pain Headache Reports.* **25**, 21. 10.1007/s11916-021-00938-3 (2021). 10.1007/s11916-021-00938-3 PMC7946573 33693989

[7] Pearlman, O. Reviewing the use of glutaraldehyde for high-level disinfection by sonographers. *J. Diagn. Med. Sonogr.* **35**(1), 49–57. 10.1177/8756479318813361 (2019).

[8] Jiang, X. L. et al. The long-term effect of glutaraldehyde on the bacterial community in anaerobic ammonium oxidation reactor. *J. Bioresource Technol.* **385**, 129448. 10.1016/j.biortech.2023.129448 (2023). 10.1016/j.biortech.2023.129448 37399960

[9] Takigawa, T. & Endo, Y. Effects of glutaraldehyde exposure on human health. *J. Occup. Health.* **48**(2), 75–87 (2006). 16612035 10.1539/joh.48.75

[10] Thumtecho, S., Sriapha, C., Tongpoo, A., Udomsubpayakul, U., Wananukul, W., Trakulsrichai, S. Poisoning of glutaraldehyde-containing products:clinical characteristics and outcomes. *J. Clin. Toxicol*. 10.1080/15563650.2020.183223 1ISSN: (Print) (Online) https://www.tandfonline.com/loi/ictx20 (2020) 10.1080/15563650.2020.1832231 33112670

[11] Gautam, K., Kumar, S., Srivastava, S., Singh, P. Pharmacological properties of Citrullus lanatus. B. eBook ISBN: 978–81–961090–9–7. 10.9734/bpi/pramr/v6/4105B (2023)

[12] Rajizadeh, A. M. et al. Investigating the effects of *Citrullus colocynthis* on cognitive performance and anxiety-like behaviors in STZ-induced diabetic rats. *Int. J. Neurosci*10.1080/00207454.2021.1916743 (2021). 33848216 10.1080/00207454.2021.1916743

[13] Saadatmand, M. et al. Green synthesis of zinc nanoparticles using Lavandula angustifolia Vera. Extract by microwave method and its prophylactic effects on Toxoplasma gondii infection. *Saudi J. Biol. Sci.* **28**, 6454–6460 (2021). 34764762 10.1016/j.sjbs.2021.07.007 PMC8568829

[14] Afzal, M. et al. Characterization of bioactive compounds and novel proteins derived from promising source citrulluscolocynthis along with in-vitro and in-vivo activities. *J Molecules* **28**, 1743. 10.3390/molecules28041743 (2023). 10.3390/molecules28041743 PMC9960351 36838731

[15] Bharti, D. B. & Bharati, A. V. Synthesis of ZnO nanoparticles using a hydrothermal method and a study its optical activity. *Luminescence.* **32**(3), 317–320 (2017). 27430489 10.1002/bio.3180

[16] Jin, Y. et al. Hydroponic cultured ginseng leaves zinc oxides nanocomposite stabilized with CMC Polymer for degradation of hazardous dyes in wastewater treatment. *J. Mater.* **14**, 6557. 10.3390/ma14216557 (2021). 10.3390/ma14216557 PMC8585460 34772099

[17] Abayarathne, H. M. I., Dunuweera, S. P. & Rajapakse, R. M. G. Synthesis of cisplatin encapsulated zinc oxide nanoparticles and their application as a carrier in targeted drug delivery. *Ceylon J. Sci.* **49**(1), 71–79. 10.4038/cjs.v49i1.7707 (2020).

[18] Breysse, P.N. Toxicological Profile for Glutaraldehyde. Book. ATSDR The ATSDR toxicological profile. http://www.atsdr.cdc.gov (2017)

[19] Ali, A. W. et al. Protective effect of rutin and β-cyclodextrin against hepatotoxicity and nephrotoxicity induced by lambda-cyhalothrin in Wistar rats: Biochemical, pathological indices and molecular analysis. *J. Biomarkers* **27**(7), 625–636. 10.1080/1354750X.2022.2087003 (2022). 10.1080/1354750X.2022.2087003 35658761

[20] Bancroft, J. D. *Theory and Practice of Histological Techniques* 6th edn. (Elsevier Heal Sci, 2008).

[21] Aziz, R. L. A. et al. Physiological roles of propolis and red ginseng nanoplatforms in alleviating dexamethasone-induced male reproductive challenges in a rat model. *Mol. Biol. Rep.* **51**(1), 72. 10.1007/s11033-023-08991-4 (2024). 38175282 10.1007/s11033-023-08991-4 PMC10766727

[22] Abdelrahman, R. E. et al. Quercetin ameliorates ochratoxin A-Induced immunotoxicity in broiler chickens by modulation of PI3K/AKT pathway. *Chem. Biol. Interact.* **351**, 109720. 10.1016/j.cbi.2021.109720 (2022). 34717913 10.1016/j.cbi.2021.109720

[23] AbdElrazek, D. A. et al. Ameliorative effects of rutin and rutin-loaded chitosan nanoparticles on testicular oxidative stress and histological damage induced by cyclophosphamide in male rats. *Food Chem. Toxicol.* **184**, 114436. 10.1016/j.fct.2024.114436 (2024). 38211767 10.1016/j.fct.2024.114436

[24] Virji, M. A., Bowers, L. N. & LeBouf, R. F. Inhalation and skin exposure to chemicals in hospital settings. *J. Handbook Indoor Air Qual.*10.1007/978-981-10-5155-5_60-1 (2022).

[25] Mohanta, Y. K. et al. Antimicrobial, antioxidant and cytotoxic activity of silver nanoparticles synthesized by leaf extract of *Erythrina suberosa* (Roxb.). *Front Mol Biosci.* **4**, 14 (2017). 28367437 10.3389/fmolb.2017.00014 PMC5355429

[26] Bourhia, M. et al. Chemical profiling, antioxidant, antiproliferative, and antibacterial potentials of chemically characterized extract of citrullus colocynthis L. seeds. *Separations.* **8**, 114 (2021).

[27] Rasool, F. Assessment of the hepatic effects of alcoholic extract of citrullus colocynthis on rat livers and antibacterial screening of the plant. *Am. J. Hortic. Floriculture Res*. (ISSN – 2689–0976) (2023)

[28] Khalaf, A. A. et al. Ameliorative effect of zinc oxide nanoparticles against dermal toxicity induced by lead oxide in rats. *Int. J. Nanomed.* **20**(14), 7729–7741. 10.2147/IJN.S220572.PMID:31806958;PMCID:PMC6855620 (2019). 10.2147/IJN.S220572 PMC6855620 31806958

[29] Azizi, S., Mohamad, R. & Shahri, M. M. Green microwave-assisted combustion synthesis of zinc oxide nanoparticles with Citrullus colocynthis (L.) Schrad: Characterization and biomedical applications. *J. Mol.* **22**, 301. 10.3390/molecules22020301 (2017). 10.3390/molecules22020301 PMC6155814 28212344

[30] Patel, P., Garala, K., Singh, S., Prajapati, B. G. & Chittasupho, C. Lipid-based nanoparticles in delivering bioactive compounds for improving therapeutic efficacy. *J. Pharmac.* **17**, 329. 10.3390/ph17030329 (2024). 10.3390/ph17030329 PMC10975431 38543115

[31] Morgan, A. M. et al. The ameliorative effect of N-acetylcysteine against penconazole induced neurodegenerative and neuroinflammatory disorders in rats. *J. Biochem. Mol. Toxicol.* **35**(10), e22884. 10.1002/jbt.22884 (2021). 34392569 10.1002/jbt.22884

[32] Rutala, W. A. & Weber, D. J. Disinfection, sterilization and antisepsis: an overview. *Am. J. Infect. Control.* **47**, A3–A9 (2019). 10.1016/j.ajic.2019.01.018 31146848

[33] Alzarah, I. A., Althobiati, F., Abbas, O. A., Mehaisen, G. M. & Kamel, N. N. Citrullus colocynthis seeds: A potential natural immune modulator source for broiler reared under chronic heat stress. *J. Animals.* **11**, 1951. 10.3390/ani11071951 (2021). 10.3390/ani11071951 PMC8300381 34208851

[34] El-Kady, A. M. H., Hassan, A. H., Mohamed, M. A. A., Malak, G. L. & Abd El-Ghaffar, K. S. Therapeutic pharmacological properties of Citrullus colocynthis fruit pulps methanolic crude extract against potassium oxonate-induced hyperuricemic gout rat model. *J. Bull. Pharm. Sci.* **46**(1), 449–463 (2023).

[35] Goma, A. A. et al. Examining the influence of zinc oxide nanoparticles and bulk zinc oxide on rat brain functions: a comprehensive neurobehavioral, antioxidant, gene expression, and histopathological investigation. *J. Biol. Trace Element Res.* **202**, 4654–4673. 10.1007/s12011-023-04043-x13 (2024). 10.1007/s12011-023-04043-x PMC11339107 38190061

[36] Pandey, P., Bharti, R. & Jasrasaria, N. Glutaraldehyde-induced allergic contact dermatitis: a case report and safety standards. *Cureus* **16**(3), e56954. 10.7759/cureus.56954 (2024). 38665736 10.7759/cureus.56954 PMC11044843

[37] Roy, R., Singh, S. K., Das, M., Tripathi, A. & Dwivedi, P. D. Toll-like receptor 6 mediated inflammatory and functional responses of zinc oxide nanoparticles primed macrophages. *Immunology* **142**, 453–464 (2014). 24593842 10.1111/imm.12276 PMC4080961

[38] Ye, K., Huang, M., He, X., An, Z. & Qin, H. Synergistic antibacterial effect of zinc oxide nanoparticles and polymorphonuclear neutrophils. *J. Funct. Biomater.* **13**, 35. 10.3390/jfb13020035 (2022). 35466217 10.3390/jfb13020035 PMC9036266

[39] Elbatouti, G. A., Abdelhady, S. A., Yacout, D. M., Farrage, E. & Abdelwahab, I. A. Evaluation of wound healing parameters and antibacterial effect of Jojoba and Citrullus colocynthis oils in staphylococcus wound infection induced in mice. *J. Pure Appl. Microbiol.* **17**(2), 1255–1265. 10.22207/JPAM.17.2.01 (2023).

[40] Mazher, M. et al. Biosynthesis and characterization of calcium oxide nanoparticles from Citrullus colocynthis fruit extracts their biocompatibility and bioactivities. *J. Mater.* **16**, 2768. 10.3390/ma16072768 (2023). 10.3390/ma16072768 PMC10096045 37049061

[41] Jakovija, A. The immune response in wound healing and cancer. UNSW Faculty ScienceMedicine & Health. 10.26190/unsworks/25344 (2023)

[42] Itoh, T. et al. Phenolic glycosides citrulluside H and Citrulluside t isolated from young watermelon (Citrullus lanatus) fruit have beneficial effects against cutibacterium acnes-induced skin inflammation. *Nat. Product Commun.* **18**(1), 1–11. 10.1177/1934578X221143202 (2022).

[43] Feng, H. et al. Dual function of peroxiredoxin I in lipopolysaccharide-induced osteoblast apoptosis via reactive oxygen species and the apoptosis signal-regulating kinase 1 signaling pathway. *Cell Death Dis.* **4**, 47. 10.1038/s41420-018-0050-9 (2018). 10.1038/s41420-018-0050-9 PMC5919897 29707240

[44] Hassanen, E. I. et al. Ameliorative effect of ZnO-NPs against bioaggregation and systemic toxicity of lead oxide in some organs of albino rats. *Environ Sci Pollut Res Int.* **28**(28), 37940–37952. 10.1007/s11356-021-13399-3 (2021). 33723775 10.1007/s11356-021-13399-3

[45] Xie, J. et al. Recent advances in ZnO nanomaterial-mediated biological applications and action mechanisms. *J. Nanomaterials.* **13**, 1500. 10.3390/nano13091500 (2023). 10.3390/nano13091500 PMC10180283 37177043

[46] Abd El-Baset, S. A., Mazen, N. F., Abdul-Maksoud, R. S. & Kattaia, A. A. A. The therapeutic prospect of zinc oxide nanoparticles in experimentally induced diabetic nephropathy. *Tissue Barriers.* **11**, 2069966 (2022). 35504734 10.1080/21688370.2022.2069966 PMC9870014

[47] Arwal, H. & Shanmugam, V. A review on anti-inflammatory activity of green synthesized zinc oxide nanoparticle: Mechanismbased approach. *Bioorg. Chem.* **94**, 103423 (2020). 31776035 10.1016/j.bioorg.2019.103423

